# 19 Channel Z-Score and LORETA Neurofeedback: Does the Evidence Support the Hype?

**DOI:** 10.1007/s10484-018-9420-6

**Published:** 2018-09-25

**Authors:** Robert Coben, D. Corydon Hammond, Martijn Arns

**Affiliations:** 1 Integrated Neuroscience Services, 92 W. Sunbridge Drive, Fayetteville, AR 72701 USA; 2 University of Utah School of Medicine, Salt Lake City, UT USA; 3 Department of Experimental Psychology, Utrecht University, Utrecht, The Netherlands; 4 Research Institute Brainclinics, Nijmegen, The Netherlands; 5 neuroCare Group, Munich, Germany

**Keywords:** LORETA neurofeedback, Z-Score neurofeedback, Review, Multichannel neurofeedback

## Abstract

Neurofeedback is a well-investigated treatment for ADHD and epilepsy, especially when restricted to standard protocols such as theta/beta, slow cortical potentials and sensori-motor rhythm neurofeedback. Advances in any field are welcome and other techniques are being pursued. Manufacturers and clinicians are marketing ‘superior’ neurofeedback approaches including 19 channel Z-score neurofeedback (ZNFB) and 3-D LORETA neurofeedback (with or without Z-scores; LNFB). We conducted a review of the empirical literature to determine if such claims were warranted. This review included the above search terms in Pubmed, Google scholar and any references that met our criteria from the ZNFB publication list and was restricted to group based studies examining improvement in a clinical population that underwent peer review (book chapters, magazine articles or conference presentations are not included since these are not peer reviewed). Fifteen relevant studies emerged with only six meeting our criterion. Based on review of these studies it was concluded that empirical validation of these approaches is sorely lacking. There is no empirical data that supports the notion that 19-channel z-score neurofeedback is effective or superior. The quality of studies for LNFB was better compared to ZNFB and some suggestion for efficacy was demonstrated for ADHD and Tinnitus distress. However, these findings need to be replicated, extended to other populations and have yet to show any “superiority.” Our conclusions continue to emphasize the pervasive lack of evidence supporting these approaches to neurofeedback and the implications of this are discussed.

## Introduction

Neurofeedback is a well-investigated treatment for ADHD and epilepsy, especially when restricted to standard protocols such as theta/beta (TBR), slow cortical potentials (SCP) and sensori-motor rhythm (SMR) neurofeedback (Arns et al. 2014). This has become evident from several meta-analyses (Arns et al. 2009; Micoulaud-Franchi et al. 2014), including a critical meta-analysis from the European ADHD Guidelines Group (EAGG) that also conducted a sensitivity analysis focused on so called ‘blinded’ ratings and hence focused on teacher reports only (Cortese et al. 2016). This latter meta-analysis did not find an overall effect of neurofeedback on teacher rated ADHD symptoms, but only when restricting the analysis to the above mentioned ‘standard protocols’ (Cortese et al. 2016). In addition to the meta-analytical support above and only focusing on randomized controlled trials (RCT’s) for the treatment of ADHD there is evidence for clinical efficacy of standard neurofeedback protocols from three large multicenter RCT’s (Gevensleben et al. 2009; Steiner et al. 2014; Strehl et al. 2017); non-superiority of methylphenidate relative to neurofeedback (Duric et al. 2012; Meisel et al. 2013) and sustained effects of neurofeedback from 3 to 9 months follow-up (Van Doren et al. 2018; Heinrich et al. 2004; Strehl et al. 2006; Leins et al. 2007; Gevensleben et al. 2010; Duric et al. 2012; Li et al. 2013; Meisel et al. 2013; Christiansen et al. 2014; Steiner et al. 2014; Bink et al. 2016) to 2 years follow-up (Gani et al. 2008). In line with the guidelines for rating evidence developed by the APA, ‘standard’ neurofeedback protocols have been considered to be ‘Efficacious and Specific, Level V’ in the treatment of ADHD (AAPB guidelines: Arns et al. 2016).

There is also a lineage of research and support for the use of SMR training, and to some degree SCP, in the treatment of seizure disorders (Sterman and Egner 2006; Tan et al. 2009). In addition there is also growing evidence to support standard protocols (1–2 channel) applied to autism, sleep, head injury and learning disorders (For review see: Tan et al. 2016), albeit to date the strongest evidence for efficacy exists for ADHD. Controlled studies have provided some additional empirical support for the use of two channel coherence training for head injury, autism and learning disabilities (Coben and Myers 2010; Coben et al. 2014, 2015; Thornton and Carmody 2005).

This notion of various types of neurofeedback protocols as well as various neurofeedback implementations is an important topic causing a lot of controversy in the field of psychiatry and neuromodulation. To most people, neurofeedback is regarded as the unitary phenomenon of neurofeedback, implying that all neurofeedback implementations are ‘thought’ to be the same, and hence combined into one meta-analysis or review. However, neurofeedback should be regarded as an umbrella term, incorporating an almost infinite number of possibilities for protocols and implementations, much similar to the umbrella term ‘medication’ which includes many different classes of medications (i.e. antidepressants, antipsychotics, antibiotics, pain-killers etc.) as well as implementations (e.g. oral dispension, intra-venously, intra-muscular etc.). The fact that it is now known that some specific neurofeedback protocols e.g. posterior alpha training in ADHD, (Nall 1973) and implementations e.g. entertainment-like playstation feedback (Arnold et al. 2013; DeBeus and Kaiser 2011) do not work in the treatment of ADHD, actually implies ‘specificity’ for some neurofeedback protocols and further supports the notion to look in more detail into the specifics of different neurofeedback protocols and implementations. For further discussions on appropriate neurofeedback implementations e.g. learning theory and conditioning, the interested reader is also referred to Sherlin et al. (2011), Arns et al. (2014) and Strehl (2014).

Medical devices in the US are regulated by the Federal Drug Administration (FDA), who can grant FDA marketing approval to specific devices. This procedure consists of companies providing their own data to the FDA for inspection and when FDA marketing approval is granted, such a company can use that claim in their marketing and selling of the device. Unfortunately, for biofeedback and neurofeedback there are no FDA marketing approvals other than the ‘exempt’ category of biofeedback for relaxation (under neurological devices, 882.5050). On the other hand, FDA marketing approval does not reflect the actual level of evidence for a given approach as accepted and endorsed in fields such as psychology or psychiatry (i.e. EMDR, mindfulness etc. are not FDA approved, yet widely used) and some FDA marketing approvals are not in line with actual evidence levels since only data provided by the manufacturer are inspected (e.g. the example of the theta/beta ratio as a ‘diagnostic test’ in ADHD, also see Arns et al. 2016). Therefore, officially, no clinical claims can be made associated with neurofeedback devices, although the scientific evidence for ‘standard neurofeedback protocols’ are more widely accepted these days as explained above. Yet, many manufacturers and clinicians are marketing ‘superior’ neurofeedback approaches. Several examples of this are Z-score 19 channel neurofeedback, 3-D LORETA neurofeedback (with or without Z-scores), neuroptimal and infra-slow neurofeedback. Proponents of these latter approaches often claim that no research is needed to support the efficacy of their equipment, since it is only intended for non-clinical use (neuroptimal) and thereby fall outside the scope of the FDA. However, the former approaches take a different approach, where they imply that the approach is backed by a wealth of scientific studies. Proponents of these approaches (19 channel Z-score and LORETA training) have suggested advantages over more traditional forms of neurofeedback. It is all too common for them to suggest that such full cap training reduces the number of sessions required for effective intervention (Krigbaum and Wigton 2015; Simkin et al. 2014). In the extreme, some allude to it’s superiority and suggest that it is the best and most “scientific” neurofeedback option available. For example, “…Deep brain (LORETA) neurofeedback is the most flexible and precise brain training tool available. Better imaging means the training is more specific to your goals, and fewer sessions are required to see results. That is why the vast majority of experienced clinicians (10 years plus) use this method of neurofeedback in their clinics… LORETA Z-Score neurofeedback is FDA approved, and meets the highest criteria for evidence-based neurofeedback practice. It has been cross-validated by both PET and MRI, is used by Universities, Medical Centers, the US military, Research Institutes, and in over 650 neurofeedback studies...” (http://www.brainworksneurotherapy.com/deep-brain-neurofeedback). Such claims sound really promising and exciting and thus were the primary aim to undertake this systematic review, to investigate the scientific evidence base behind these claims.

In line with what was stated earlier, it should be noted that LORETA Z-score neurofeedback is not “FDA approved”, other then maybe registered at the FDA under the exempt category.

Below we will summarize these approaches in a bit more detail to familiarize the reader with these methods.

### Z-Score Neurofeedback (ZNFB)

One of the initial descriptions of 19 channel ZNFB training can be found in Collura et al. (2009). The approach includes applying live z-score training across all 19 surface channels using joint time frequency analysis using a QEEG database (i.e., Neuroguide, BrainDx). One may use any number of targeting approaches such as training amplitude, power ratios, coherence, or a combination of targets. The goal of training is for the client to move towards a z = 0 or ‘normalcy’ compared to a QEEG database. It has been claimed that using this approach leads to effective clinical outcomes in fewer sessions than traditional neurofeedback (Krigbaum and Wigton 2015).

### LORETA Neurofeedback (LNFB)

LORETA is a popular inverse solution technique that estimates the three-dimensional origination of electrical signals based on a grid of electrodes placed all over the scalp (Pascual-Marqui et al. 1994). Congedo et al. (2004) initially presented using this current source density calculation as the basis for an approach to neurofeedback. Several well-conducted proof-of-concept studies on this method have been pioneered by Marco Congedo and Rex Cannon (See Cannon et al. 2007; Congedo et al. 2004), however in this systematic review we mainly intend to summarize the evidence of the clinical applications of these methods. Other applications of this approach have combined it with the use of live z scores for training into what is now known as LORETA ZNFB (Thatcher 2013). In this approach it is theorized that the neurofeedback is training deeper sources of the EEG than can be accomplished by training at the surface with a small number of electrodes.

These approaches do represent innovations compared to other more traditional forms of neurofeedback and aim to get similar improved signal:noise ratio’s and spatial resolution as is seen with fMRI-neurofeedback.

## Methods

We conducted a systematic literature review using the search terms “LORETA neurofeedback” and “19 channel ZNFB” in both PubMed and google scholar as of the beginning of June 2017. PubMed automatically searches the Medline database of the US National Library of Medicine with references dating back to 1996. Google scholar (https://scholar.google.com/) is a freely accessible web search engine that indexes metadata of scholarly literature across an array of publishing formats and disciplines (not just medical fields). This includes peer reviewed journals, books, conference papers abstracts, etc. As such, it provides a greater breadth of references that go beyond PubMed and includes publications in neurofeedback and neuromodulation journals and books. Google scholar began in 2004 and indexes references from this year forward. We also included in this review a list of ZNFB publications made available at the applied neuroscience website (http://appliedneuroscience.com/Z_Score_publications.pdf).

These sources were reviewed for empirical research designs that were published in peer reviewed journals and directly measured the efficacy of one of these approaches in a clinical population. Due to the scarcity of literature, the requirement for randomized controlled studies (RCT’s) was dropped rather early. Papers were only included in this review when they met the following criteria:

1. Group-based studies: case studies and case series are not included
2. Studies investigating the clinical use of neurofeedback in a specific disorder with a specifically defined primary endpoint of clinical improvement [i.e. symptom improvement defined by a valid rating scale e.g. BDI, ADHD-RS etc; proof of concept studies demonstrating a specific region of interest (ROI) can be trained were beyond the scope of this review]
3. Peer-reviewed articles in scientific journals: book chapters, magazine articles or conference presentations are not included since these are not peer reviewed. Furthermore, articles should have been published in journals that engage in adequate peer-review and thus journals that were listed on ‘Beall’s list of predatory journals and publishers’ (https://beallslist.weebly.com) were excluded (for review of predatory journals see Butler 2013)

## Results

The PubMed search under the term LNFB resulted in five studies, and under 19 channel ZNFB four studies. The additional Google scholar search attracted six additional LNFB studies and no new 19 channel ZNFB papers. Searching the applied neuroscience list of ZNFB publications resulted in no additions to our list. There are a total of 15 research studies that were reviewed in detail and form the basis of our empirical review. Of these 15, only six met the criteria listed above. Of the nine that were excluded, five included only nonclinical subjects and were considered proof of concept studies and did not test clinical efficacy. The remaining four were either case studies (YuLeung To et al. 2016), case series (Koberda et al. 2012), or not a study at all but a discussion of related topics (Cannon 2012; Simkin et al. 2014). The overall quality of these six studies were judged based on the inclusion of comparison or control groups in which subjects received an experimental manipulation that was not z-score or LNFB training. Only three such studies were found. Random assignment of subjects (another indicator of a higher quality research design) was used in only one of these.


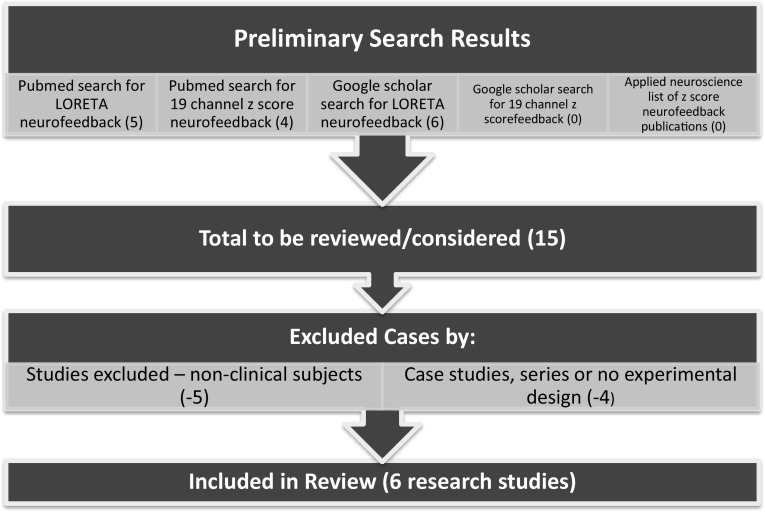


Six studies were included that fulfilled our inclusion criteria. These are listed in Table 1 above. Three of these focused on surface ZNFB. Krigbaum and Wigton (2015) sought to demonstrate a method of monitoring progress in ten cases receiving 19 channel ZNFB. They showed an improvement towards the mean in 90% of the cases in 15 or fewer sessions. Wigton and Krigbaum (2015) conducted a pilot study of 21 subjects where they assessed neuropsychological and behavioral rating measures on about half of those subjects. Most subjects had ADHD/ADD, albeit no diagnostic information is provided, nor supportive details of diagnosis. There were significant pre- to post-training changes on all measures, but no comparison group was included for comparison and none of the outcome metrics assessed, allowed a comparison to prior meta-analysis (e.g. no DSM based ADHD-RS data). Hammer et al. (2011) conducted a study to investigate if ZNFB could help a group with insomnia. Twelve adults with insomnia met their inclusion criterion, but only eight completed the training. These subjects were divided into two groups, including a four channel ZNFB surface group and a two channel (Cz, C4) ZNFB SMR training group. Sleep measures improved in both groups and there was no difference between the two groups in terms of outcome. There have been no studies using 19 channel ZNFB published in peer reviewed journals that have included any comparison group to understand how this form of neurofeedback might compare to control conditions (not even a wait list control group) or (semi-)active treatments.

**Table 1:** Review of empirical studies

References	Clinical group (number of sessions)	Total number of subjects across groups	Control group comparisons	Random assignment	Targeted QEEG change
Hammer et al. (2011)	Sleep disorders (15 sessions)	N = 8	Two treatment groups (both z-score)	Yes	No difference between the groups
Liechti et al. (2012)	ADHD (36 sessions)	N = 13 in the tNF group	Two comparison groups (single channel neurofeedback and EMG biofeedback training groups)	Yes	No
Cannon et al. (2014)	Mixed control and clinical population (10–20 sessions)	N = 13	One comparison group	No	Not in the clinical group
Krigbaum and Wigton (2015)	Mixed clinical population (15 or fewer session)	N = 10	None	No	Yes
Wigton and Krigbaum (2015)	Mixed clinical population	N = 21	None	No	Yes
Vanneste et al. (2016)	Tinnitus (15 sessions)	N = 58	Two comparison groups (two locations of LNFB training and a wait list control group)	No	Not in LORETA frequency domain or ROI, but positive changes were seen in measures of connectivity

Only three research studies have evaluated if LNFB has a significant impact on clinical populations compared to appropriate comparison groups. Liechti et al. (2012) have conducted the only controlled trial comparing the effects of LNFB in children with ADHD. Subjects were randomly assigned to a LNFB, standard neurofeedback (Cz) or EMG biofeedback condition, with 14 total participants in the LNFB group. In this latter group they conducted theta-beta and SCP training in counterbalanced order. Symptom ratings decreased significantly in the LNFB group over a span of 36 sessions with effect sizes (ES) within the range of ES published before for ‘standard neurofeedback protocols’ in ADHD (Parent rated inattention ES = 1.26; Hyperactivity/Impulsivity ES = 0.65). However, learning to control the targeted region (anterior cingulate) in the LNFB group was limited. Rather, they demonstrated good control of artifact, eye movement and muscle activity. While there were control conditions in this research, they were not directly compared in this study. The authors concluded that the effects could be from artifact reduction and not the direct effects of the brain regions targeted. In addition, the number of training sessions was approximately the number of sessions commonly seen in traditional neurofeedback research with ADD/ADHD and ES comparable to studies using ‘standard protocols’.

More recently, Cannon et al. (2014) studied the effects of LNFB training in the precuneus. Thirteen participants received 10–20 sessions of training over the left precuneus, including 5 healthy students and 8 subjects with a variety of psychiatric diagnoses. Outcome was further assessed with the Personality Assessment Inventory and Delis-Kaplan Executive Functioning System. Their findings showed significant decreases on PAI scales of psychological distress, but not as much on the cognitive processing measures. Also, they were able to show ROI EEG changes in the precuneus of the controls but not in the clinical subjects.

In another higher quality study, Vanneste et al. (2016) researched the effects of LNFB on tinnitus related distress. A total of 58 subjects were included, 23 received LNFB of the posterior cingulate (alpha up and beta down), 17 LNFB of the lingual gyrus and 18 were part of a wait list control group. The findings showed no significant change on tinnitus loudness, but distress was diminished in the posterior cingulate trained group with a large ES (ES = 0.70) but not for the other groups. This effect was explained by a select number in that group; close to half of those with grade iv distress moved to a lower level of distress. Analysis of the brain change data showed no change of the targeted activity at specific regions, but rather a decrease in connectivity between the posterior cingulate and dorsal anterior cingulate. These findings are fascinating, also due to the protocol specific effects and did include appropriate control groups.

## Discussion

Progress in the field of neurofeedback has been stunted by premature claims of efficacy that are not accepted by those outside of our field. For this reason, it is crucial to be clear about what the empirical evidence says about our approaches and techniques (see Coben and Evans 2010).

From a theoretical perspective, these methods that include a greater number electrodes, train towards normality and involve source localized activity as their target would represent methodological and conceptual enhancements. Congedo et al. (2004) were able to design and demonstrate the first application of LNFB which they used in a non-clinical population. These subjects were able to learn volitional control over this feedback signal. Similarly, Cannon and his colleagues have used LNFB in samples of non-clinical subjects to demonstrate its utility. Cannon et al. (2006) were able to target the cognitive division of the anterior cingulate gyrus leading to enhancements in attention and working memory in college students. In a subsequent project, they showed that training led to changes in the anterior cingulate (Cannon et al. 2007), which was targeted, but also to other frontal and even parietal regions as well. Interestingly, Getter et al. (2015) was able to show in only two cases that training over parietal regions altered a mental rotation task (related to parietal functioning). So, while these experimental manipulations and demonstrations are interesting, they are not a demonstration of clinical efficacy which requires specific research findings in clinical subjects with valid clinical outcome measures and control groups.

This review of empirical studies shows that such information is sorely lacking. For ZNFB there is virtually no evidence that it is effective nor that it is “superior” in any way to other forms of neurofeedback. For ZNFB none of the studies included an empirical comparison to any other type of control or treatment group in a clinical population and also none of the outcome measures allowed comparison to prior meta-analysis. As a result, it must be considered baseless in it’s demonstration of clinical efficacy. The quality of studies for LNFB was better compared to ZNFB and some suggestion for efficacy was demonstrated for ADHD (Liechti et al. 2012) and Tinnitus (Vanneste et al. 2016), albeit none of the findings have been replicated and the ES for ADHD was similar to those reported before for ‘traditional neurofeedback protocols’ (Arns et al. 2009). In fact, these effects were hypothesized to be related to control external artifact. For tinnitus, no effects were found for tinnitus loudness, whereas a protocol specific effect was found for distress with a large effect size. Replication and expansion of these findings would clearly be important. Furthermore, LNFB did not show ROI EEG changes in clinical subjects in Cannon et al. (2014) analyses. In light of these findings ZNFB and LNFB protocols cannot be recommended as a first line treatment option and these protocols remain experimental. When standard neurofeedback protocols and other well-investigated treatments have not yielded the desired effects, ZNFB and LNFB could be considered as an experimental off-label treatment, if patients are sufficiently informed about the experimental nature of the protocols and informed consent is used. However, the evidence reviewed in this manuscript does not justify widely advertising ZNFB and LNFB as better and faster alternatives until more solid studies have been conducted.

Our conclusions continue to emphasize the pervasive lack of evidence supporting these approaches to neurofeedback. It is interesting in this light that Koberda et al. (2012) show very little change in efficacy moving from one channel to LNFB. Also, Cannon (2012) discussed possible adverse effects for specific LNFB protocols. We would recommend considering these approaches as potentially promising with a great need for more research prior to making claims of efficacy and certainly could not endorse the notion of their superiority. We strongly encourage more research be devoted to these approaches across multiple clinical conditions and would be supportive with further evidence supporting efficacy and safety. Until then, these should be considered experimental.

## References

[CR1] Arnold LE, Lofthouse N, Hersch S, Pan X, Hurt E, Bates B (2013). EEG neurofeedback for ADHD: Double-blind sham-controlled randomized pilot feasibility trial. Journal of Attention Disorders.

[CR2] Arns M, de Ridder S, Strehl U, Breteler M, Coenen A (2009). Efficacy of neurofeedback treatment in ADHD: The effects on inattention, impulsivity and hyperactivity: A meta-analysis. Clinical EEG and Neuroscience.

[CR3] Arns M, Heinrich H, Strehl U (2014). Evaluation of neurofeedback in ADHD: The long and winding road. Biological Psychology.

[CR4] Arns, M., Heinrich, H., & Strehl, U. (2016). Attention deficit hyperactivity disorder (ADHD) level 5: Efficacious and specific. In *Evidence-based practice in biofeedback & neurofeedback* (3rd ed., pp. 18–22).

[CR5] Bink M, Bongers IL, Popma A, Janssen TW, van Nieuwenhuizen C (2016). 1-year follow-up of neurofeedback treatment in adolescents with attention-deficit hyperactivity disorder: Randomised controlled trial. BJPsych Open.

[CR6] Butler D (2013). The dark side of publishing. Nature.

[CR7] Cannon R, Lubar J, Congedo M, Thornton K, Towler K, Hutchens T (2007). The effects of neurofeedback training in the cognitive division of the anterior cingulate gyrus. International Journal of Neuroscience.

[CR8] Cannon R, Lubar J, Gerke A, Thornton K, Hutchens T, McCammon V (2006). EEG spectral-power and coherence: LORETA neurofeedback training in the anterior cingulate gyrus. Journal of Neurotherapy.

[CR9] Cannon RL (2012). LORETA neurofeedback: Odd reports, observations, and findings associated with spatial specific neurofeedback training. Journal of Neurotherapy.

[CR10] Cannon RL, Baldwin DR, Diloreto DJ, Phillips ST, Shaw TL, Levy JJ (2014). LORETA neurofeedback in the precuneus: Operant conditioning in basic mechanisms of self-regulation. Clinical EEG and Neuroscience.

[CR11] Christiansen H, Reh V, Schmidt MH, Rief W (2014). Slow cortical potential neurofeedback and self-management training in outpatient care for children with ADHD: Study protocol and first preliminary results of a randomized controlled trial. Frontiers in Human Neuroscience.

[CR12] Coben R, Evans JR (2010). Neurofeedback and neuromodulation techniques and applications.

[CR13] Coben R, Myers TE (2010). The relative efficacy of connectivity guided and symptom based EEG biofeedback for autistic disorders. Applied Psychophysiology & Biofeedback.

[CR14] Coben R, Sherlin L, Hudspeth WJ, McKeon K, Ricca R (2014). Connectivity-guided EEG biofeedback for autism spectrum disorder: Evidence of neurophysiological changes. NeuroRegulation.

[CR15] Coben R, Wright EK, Decker SL, Morgan T (2015). The impact of coherence neurofeedback on reading delays in learning disabled children: A randomized controlled study. NeuroRegulation.

[CR16] Collura, T. F., Thatcher, R. W., Smith, M. L., Lambos, W. A., & Stark, C. A. (2009). EEG biofeedback training using live Z-scores and a normative database. In *Introduction to Quantitative EEG and Neurofeedback* (pp. 103–141).

[CR17] Congedo M, Lubar JF, Joffe D (2004). Low-resolution electromagnetic tomography neurofeedback. IEEE Transactions on Neural Systems and Rehabilitation Engineering.

[CR18] Cortese S, Ferrin M, Brandeis D, Holtmann M, Aggensteiner P, Daley D (2016). Neurofeedback for attention-deficit/hyperactivity disorder: Meta-analysis of clinical and neuropsychological outcomes from randomized controlled trials. Journal of the American Academy of Child & Adolescent Psychiatry.

[CR19] DeBeus RJ, Kaiser DA (2011). Neurofeedback with children with attention deficit hyperactivity disorder: A randomized doubleblind placebo-controlled study. Neurofeedback and Neuromodulation Techniques and Applications.

[CR20] Duric NS, Assmus J, Gundersen D, Elgen IB (2012). Neurofeedback for the treatment of children and adolescents with ADHD: A randomized and controlled clinical trial using parental reports. BMC Psychiatry.

[CR21] Gani C, Birbaumer N, Strehl U (2008). Long term effects after feedback of slow cortical potentials and of theta-beta amplitudes in children with attention-deficit/hyperactivity disorder. International Journal of Bioelectromagnetics.

[CR22] Getter N, Kaplan Z, Todder D (2015). Evaluating low-resolution tomography neurofeedback by single dissociation of mental grotation task from stop signal task performance. Behavioural Brain Research.

[CR23] Gevensleben H, Holl B, Albrecht B, Schlamp D, Kratz O, Studer P (2010). Neurofeedback training for children with ADHD: 6-month follow-up of a randomised controlled trial. European Child & Adolescent Psychiatry.

[CR24] Gevensleben H, Holl B, Albrecht B, Vogel C, Schlamp D, Kratz O (2009). Is neurofeedback an efficacious treatment for ADHD? A randomised controlled clinical trial. Journal of Child Psychology and Psychiatry.

[CR25] Hammer BU, Colbert AP, Brown KA, Ilioi EC (2011). Neurofeedback for insomnia: A pilot study of z-score SMR and individualized protocols. Applied Psychophysiology and Biofeedback.

[CR26] Heinrich H, Gevensleben H, Freisleder FJ, Moll GH, Rothenberger A (2004). Training of slow cortical potentials in attention-deficit/hyperactivity disorder: Evidence for positive behavioral and neurophysiological effects. Biological Psychiatry.

[CR27] Koberda JL, Hillier DS, Jones B, Moses A, Koberda L (2012). Application of neurofeedback in general neurology practice. Journal of Neurotherapy.

[CR28] Koberda JL, Moses A, Koberda P, Koberda L (2012). Comparison of the effectiveness of z-score surface/LORETA 19-electrodes neurofeedback to standard 1-electrode neurofeedback. Journal of Neurotherapy.

[CR29] Krigbaum G, Wigton NL (2015). A methodology of analysis for monitoring treatment progression with 19-channel z-score neurofeedback (19ZNF) in a single-subject design. Applied Psychophysiology and Biofeedback.

[CR100] Leins U, Goth G, Hinterberger T, Klinger C, Rumpf N, Strehl U (2007). Neurofeedback for children with ADHD: A comparison of SCP and theta/beta protocols. Applied Psychophysiology and Biofeedback.

[CR101] Li L, Yang L, Zhuo CJ, Wang YF (2013). A randomised controlled trial of combined EEG feedback and methylphenidate therapy for the treatment of ADHD. Swiss Medical Weekly.

[CR30] Liechti MD, Maurizio S, Heinrich H, Jäncke L, Meier L, Steinhausen HC (2012). First clinical trial of tomographic neurofeedback in attention-deficit/hyperactivity disorder: Evaluation of voluntary cortical control. Clinical Neurophysiology.

[CR31] Meisel V, Servera M, Garcia-Banda G, Cardo E, Moreno I (2013). Neurofeedback and standard pharmacological intervention in ADHD: A randomized controlled trial with six-month follow-up. Biological Psychology.

[CR32] Micoulaud-Franchi JA, Geoffroy PA, Fond G, Lopez R, Bioulac S, Philip P (2014). EEG neurofeedback treatments in children with ADHD: An updated meta-analysis of randomized controlled trials. Frontiers in Human Neuroscience.

[CR33] Nall A (1973). Alpha training and the hyperkinetic child-is it effective?. Academic Therapy.

[CR34] Pascual-Marqui JRD, Michel CM, Lehmann D (1994). Low resolution electromagnetic tomography: A new method for localizing electrical activity in the brain. International Journal of Psychophysiology.

[CR35] Sherlin LH, Arns M, Lubar J, Heinrich H, Kerson C, Strehl U (2011). Neurofeedback and basic learning theory: Implications for research and practice. Journal of Neurotherapy.

[CR36] Simkin DR, Thatcher RW, Lubar J (2014). Quantitative EEG and neurofeedback in children and adolescents: Anxiety disorders, depressive disorders, comorbid addiction and attention-deficit/hyperactivity disorder, and brain injury. Child and Adolescent Psychiatric Clinics of North America.

[CR37] Steiner NJ, Frenette EC, Rene KM, Brennan RT, Perrin EC (2014). In-school neurofeedback training for ADHD: Sustained improvements from a randomized control trial. Pediatrics.

[CR38] Sterman MB, Egner T (2006). Foundation and practice of neurofeedback for the treatment of epilepsy. Applied Psychophysiology and Biofeedback.

[CR39] Strehl U (2014). What learning theories can teach us in designing neurofeedback treatments. Frontiers in Human Neuroscience.

[CR40] Strehl U, Aggensteiner P, Wachtlin D, Brandeis D, Albrecht B, Arana M (2017). Neurofeedback of slow cortical potentials in children with attention-deficit/hyperactivity disorder: A multicenter randomized trial controlling for unspecific effects. Frontiers in Human Neuroscience.

[CR41] Strehl U, Leins U, Gopth G, Klinger C, Hinterberger T, Birbaumer N (2006). Self-regulation of slow cortical potentials: A new treatment for children with attention-deficit/hyperactivity disorder. Pediatrics.

[CR42] Tan G, Shaffer F, Lyle R, Teo I (2016). Evidence-based practice in biofeedback and neurofeedback.

[CR43] Tan G, Thornby J, Hammond DC, Strehl U, Canady B, Arnemann K, Kaiser DK (2009). Meta-analysis of EEG biofeedback in treating epilepsy. Clinical EEG & Neuroscience.

[CR44] Thatcher RW (2013). Latest developments in live z-score training: Symptom check list, phase reset, and LORETA z-score biofeedback. Journal of Neurotherapy.

[CR102] Thornton KE, Carmody DP (2005). Electroencephalogram biofeedback for reading disability and traumatic brain injury. Child and Adolescent Psychiatric Clinics of North America.

[CR103] Van Doren J, Arns M, Heinrich H, Vollebregt MA, Strehl U, Loo SK (2018). Sustained effects of neurofeedback in ADHD: A systematic review and meta-analysis. European Child & Adolescent Psychiatry.

[CR45] Vanneste S, Joos K, Ost J, De Ridder D (2016). Influencing connectivity and cross-frequency coupling by real-time source localized neurofeedback of the posterior cingulate cortex reduces tinnitus related distress. Neurobiology of Stress.

[CR46] Wigton NL, Krigbaum G (2015). Attention, executive function, behavior, and electrocortical function, significantly improved with 19-channel z-score neurofeedback in a clinical setting a pilot study. Journal of Attention Disorders.

[CR47] YuLeung To E, Abbott K, Foster DS, Helmer DA (2016). Working memory and neurofeedback. Applied Neuropsychology: Child.

